# Supplementary material for: Chromatin Compaction by Small RNAs and the Nuclear RNAi Machinery in C. elegans
Source: Sci Rep. 2019 Jun 21;9:9030. doi: 10.1038/s41598-019-45052-y (PMC6588724; doi:10.1038/s41598-019-45052-y)
Supplement: Supplementary file 2 — Supplementary Information Fields and Kennedy [file 41598_2019_45052_MOESM2_ESM.pdf]

Chromatin Compaction by Small RNAs and the Nuclear RNAi Machinery in *C. elegans*

Brandon D. Fields[1,2] and Scott Kennedy*[2]

1 Laboratory of Genetics, University of Wisconsin-Madison, Madison, WI 53706, USA

2 Department of Genetics, Harvard Medical School, Boston, MA 02115, USA

*Corresponding author: kennedy@genetics.med.harvard.edu

Department of Genetics, Harvard Medical School

77 Avenue Louis Pasteur

New Research Building 266

Boston, MA. 02215

Ph: 617-432-1235

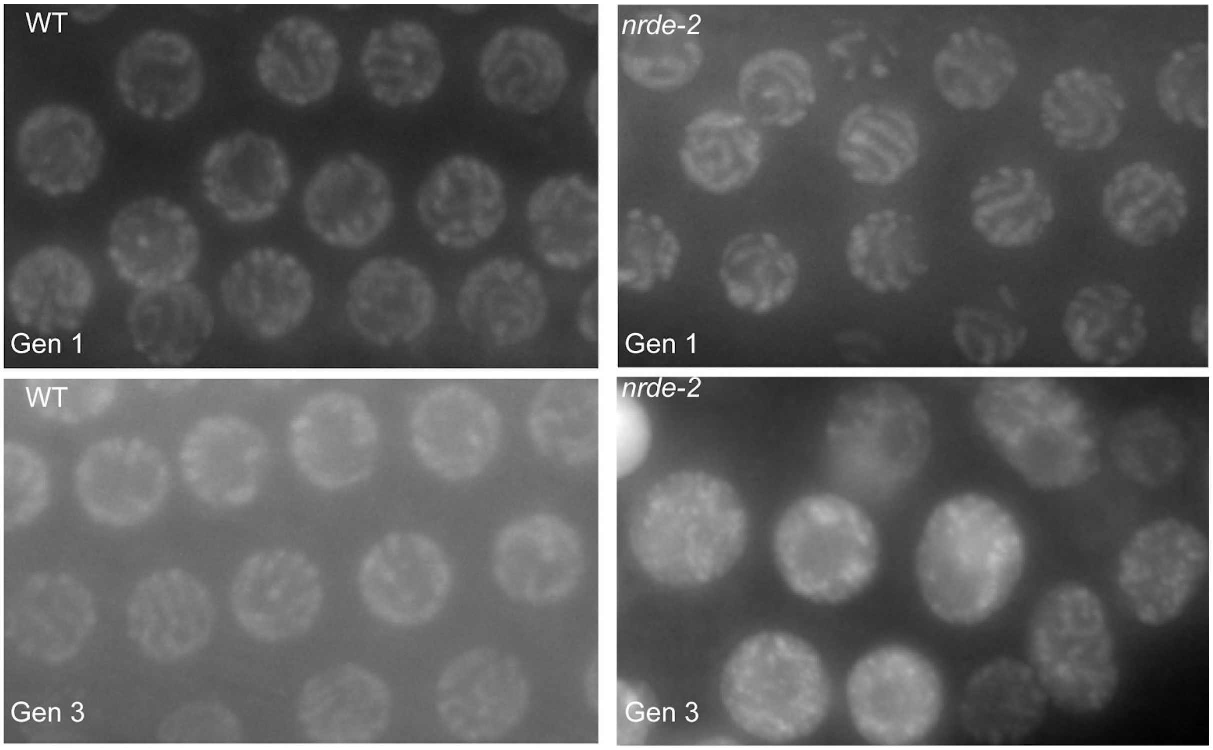

Supplementary Figure 1. Late generation *nrde-2(-)* animals display enlarged/disorganized germline chromatin. Fluorescent micrograph of pachytene germ cells of animals expressing a *gfp::h2b* transgene in the germline. Wild type or *nrde-2(gg091)* animals were maintained at 25°C for three generations. Images are to same scale.

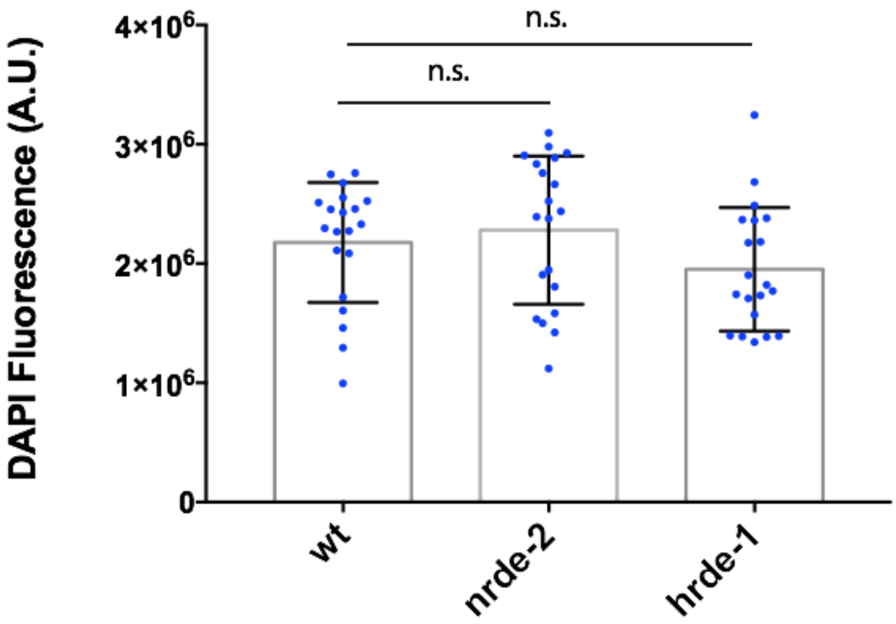

Supplementary Figure 2. Wild-type, *nrde-2(-) and hrde-1(-)* nuclei possess similar amounts of DNA. Fluorescent DAPI intensity measurements were made from individual germ cells of wild type, *nrde-2(gg091)*, and *hrde-1(tm1200)* animals grown at 25°C for three generations. Each data point represents one germ cell nuclei. Data points were collected from four different animals per genotype. p-value was calculated using a student's two tailed t-test. n.s. = not significant.

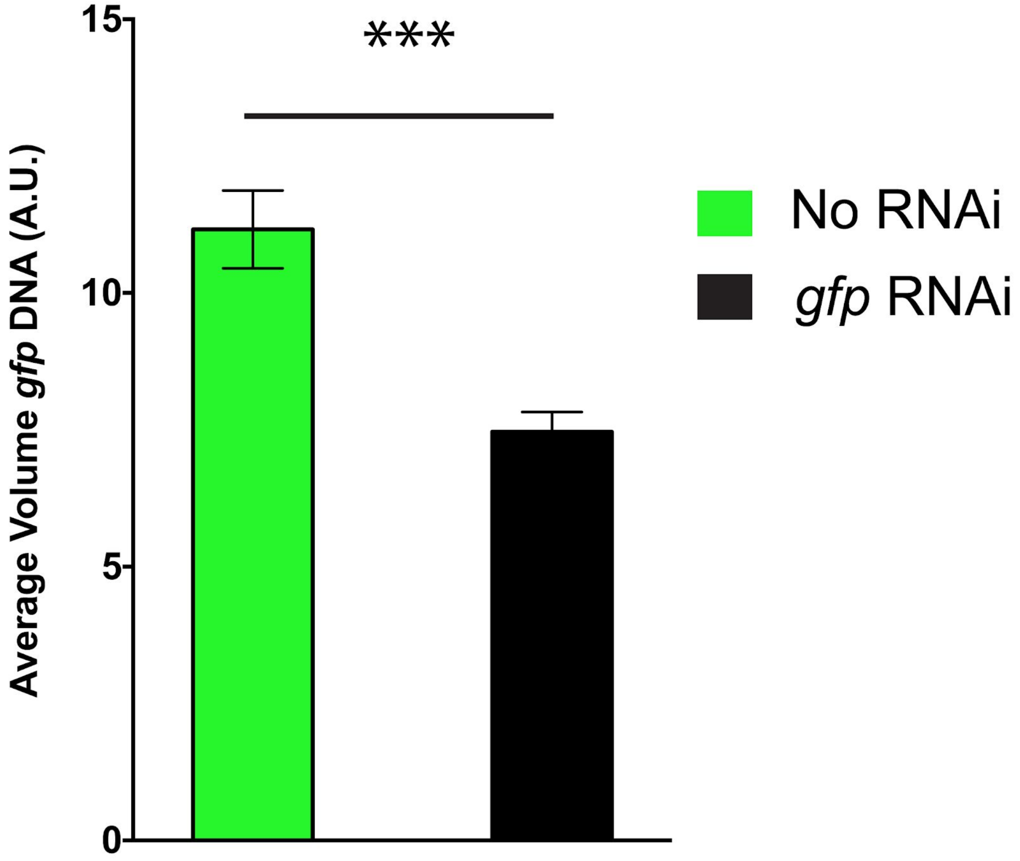

Supplementary Figure 3. Volume quantifications of the *sur-5::gfp* transgene. Volume quantifications for animals exposed to *gfp* RNAi (right) or no RNAi (left). p-value was calculated using a student’s two tailed t-test. *** = p-value <0.005.

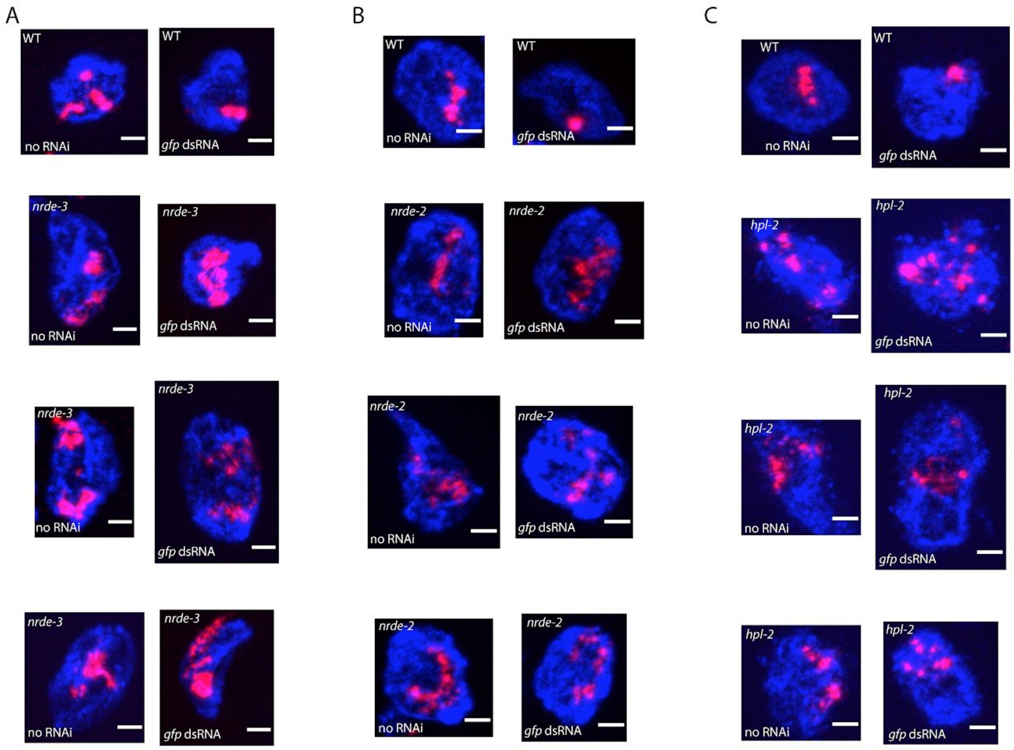

Supplementary Figure 4. Representative images of chromatin compaction in wild-type, *nrde-3*, *nrde-2*, and *hpl-2* animals. Fluorescent micrographs of intestinal nuclei from animals possessing a multicopy *sur-5::gfp* transgene stained with DNA FISH probes targeting *gfp* DNA (red) and DAPI (blue) in wild type or *nrde-3(gg066)* (A), wild type or *nrde-2(gg091)* (B), and wild type or *hpl-2(tm1489)* (C) mutant animals exposed to no RNAi (left) or *gfp* RNAi (right). Images are to same scale. Scale bars = 3 um.

Movie 1. 3D reconstructions of GFP::H2B fluorescence in wild type and *nrde-2(-)* mutant animals. 3D reconstructions of confocal Z-slices for wild type and *nrde-2(gg091)* animals expressing a *gfp::h2b* reporter in the germline and maintained at 25°C for three generations. Z-slices were gathered at 0.3 um intervals. 3D reconstructions were generated using Nikon Imaging software. Images are to same scale.

Sequence: Sup_File_1_gfp_RNAi_plasmid.dna (Circular / 3579 bp)
Features: 3 total

```
5'  AACCTGGCTTATCGAAATTAATACGACTCACTATAGGGAGACCGGCAGATCTGATATCATCGATG
...                                                                          65
3'  TTGGACCGAATAGCTTTAATTATGCTGAGTGATATCCCTCTGGCCGTCTAGACTATAGTAGCTAC
                        t7

    AATTCGAGCTCCACCGCGGTGGCGGCCGCTCTAGAACTAGTggatccaccgggcaTTTGTATAGT
                                                                             130
    TTAAGCTCGAGGTGGCGCCACCGCCGGCGAGATCTTGATCAcctaggtggcccgtAAACATATCA
                                                              < GFP

    TCATCCATGCCATGTGTAATCCCAGCAGCTGTTACAAACTCAAGAAGGACCATGTGGTCTCTCTT
                                                                             195
    AGTAGGTACGGTACACATTAGGGTCGTCGACAATGTTTGAGTTCTTCCTGGTACACCAGAGAGAA
  <                               GFP

    TTCGTTGGGATCTTTCGAAAGGGCAGATTGTGTGGACAGGTAATGGTTGTCTGGTAAAAGGACAG
                                                                             260
    AAGCAACCCTAGAAAGCTTTCCCGTCTAACACACCTGTCCATTACCAACAGACCATTTTCCTGTC
  <                               GFP

    GGCCATCGCCAATTGGAGTATTTTGTTGATAATGGTCTGCTAGTTGAACGCTTCCATCTTCAATG
                                                                             325
    CCGGTAGCGGTTAACCTCATAAAACAACTATTACCAGACGATCAACTTGCGAAGGTAGAAGTTAC
  <                               GFP

    TTGTGTCTAATTTTGAAGTTctgaaaatttaaatcagattagttagttagtaaaatcatgtttaa
                                                                             390
    AACACAGATTAAAACTTCAAgacttttaaatttagtctaatcaatcaatcattttagtacaaatt
  <                               GFP

    acttacAACTTTGATTCCATTCTTTTGTTTGTCTGCCATGATGTATACATTGTGTGAGTTATAGT
                                                                             455
    tgaatgTTGAAACTAAGGTAAGAAAACAAACAGACGGTACTACATATGTAACACACTCAATATCA
  <                               GFP

    TGTATTCCAATTTGTGTCCAAGAATGTTTCCATCTTCTTTAAAATCAATACCTTTTAACTCGATT
                                                                             520
    ACATAAGGTTAAACACAGGTTCTTACAAAGGTAGAAGAAATTTTAGTTATGGAAAATTGAGCTAA
  <                               GFP

    CTATTAACAAGGGTATCACCTTCAAACTTGACTTCAGCACctgaaaatttaaatatgtatggtta
                                                                             585
    GATAATTGTTCCCATAGTGGAAGTTTGAACTGAAGTCGTGgacttttaaatttatacataccaat
  <                               GFP

    gttagtaccgaactgtttaaacttacGTGTCTTGTAGTTCCCGTCATCTTTGAAAAATATAGTTC
                                                                             650
    caatcatggcttgacaaatttgaatgCACAGAACATCAAGGGCAGTAGAAACTTTTTATATCAAG
  <                               GFP
```

```
TTTCCTGTACATAACCTTCGGGCATGGCACTCTTGAAAAAGTCATGCcGTTTCATATGATCTGGG   715
AAAGGACATGTATTGGAAGCCCGTACCGTGAGAACTTTTTCAGTACGgCAAAGTATACTAGACCC
< GFP

TATCTcGAgAAGCATTGAACACCATAAcAGAAAGTAGTGACAAGTGTTGGctgaaaatttaaata   780
ATAGAgCTcTTCGTAACTTGTGGTATTgTCTTTCATCACTGTTCACAACCgacttttaaatttat
< GFP

atcagggttagttagtatatatatgtttaaacttacCCATGGAACAGGTAGTTTTCCAGTAGTGC   845
tagtcccaatcaatcatatatatacaaatttgaatgGGTACCTTGTCCATCAAAAGGTCATCACG
< GFP

AAATAAATTTAAGGGTAAGTTTTCCGTATGTTGCATCACCTTCACCCTCTCCACTGACAGAAAAT   910
TTTATTTAAATTCCCATTCAAAAGGCATACAACGTAGTGGAAGTGGGAGAGGTGACTGTCTTTTA
< GFP

TTGTGCCCATTAACATCACCATCTAATTCAACAAGAATTGGGACAACTCCAGTGAAAAGTTCTTC   975
AACACGGGTAATTGTAGTGGTAGATTAAGTTGTTCTTAACCCTGTTGAGGTCACTTTTCAAGAAG
< GFP

TCCTTTACTCATTTTTTTCTACCGGTACCCAATTCGCCCTATAGTGAGTCGTATTACGCGCGCTCA   1040
AGGAAATGAGTAAAAAAAGATGGCCATGGGTTAAGCGGGATATCACTCAGCATAATGCGCGCGAGT
< GFP                                t7

CTGGCCGTCGTTTTACAACGTCGTGACTGGGAAAACCCTGGCGTTACCCAACTTAATCGCCTTGC   1105
GACCGGCAGCAAAATGTTGCAGCACTGACCCTTTTGGGACCGCAATGGGTTGAATTAGCGGAACG

AGCACATCCCCCTTTCGCCAGCTGGCGTAATAGCGAAGAGGCCCGCACCGATCGCCCTTCCCAAC   1170
TCGTGTAGGGGGAAAGCGGTCGACCGCATTATCGCTTCTCCGGGCGTGGCTAGCGGGAAGGGTTG

AGTTGCGCAGCCTGAATGGCGAATGGGACGCGCCCTGTAGCGGCGCATTAAGCGCGGCGGGTGTG   1235
TCAACGCGTCGGACTTACCGCTTACCCTGCGCGGGACATCGCCGCGTAATTCGCGCCGCCCACAC

GTGGTTACGCGCAGCGTGACCGCTACACTTGCCAGCGCCCTAGCGCCCGCTCCTTTCGCTTTCTT   1300
CACCAATGCGCGTCGCACTGGCGATGTGAACGGTCGCGGGATCGCGGGCGAGGAAAGCGAAAGAA

CCCTTCCTTTCTCGCCACGTTCGCCGGCTTTCCCCGTCAAGCTCTAAATCGGGGGCTCCCTTTAG   1365
GGGAAGGAAAGAGCGGTGCAAGCGGCCGAAAGGGGCAGTTCGAGATTTAGCCCCCGAGGGAAATC
```

```
GGTTCCGATTTAGTGCTTTACGGCACCTCGACCCCAAAAAACTTGATTAGGGTGATGGTTCACGT
                                                                    1430
CCAAGGCTAAATCACGAAATGCCGTGGAGCTGGGGTTTTTTGAACTAATCCCACTACCAAGTGCA

AGTGGGCCATCGCCCTGATAGACGGTTTTTCGCCCTTTGACGTTGGAGTCCACGTTCTTTAATAG
                                                                    1495
TCACCCGGTAGCGGGACTATCTGCCAAAAAGCGGGAAACTGCAACCTCAGGTGCAAGAAATTATC

TGGACTCTTGTTCCAAACTGGAACAACACTCAACCCTATCTCGGTCTATTCTTTTGATTTATAAG
                                                                    1560
ACCTGAGAACAAGGTTTGACCTTGTTGTGAGTTGGGATAGAGCCAGATAAGAAAACTAAATATTC

GGATTTTGCCGATTTCGGCCTATTGGTTAAAAAATGAGCTGATTTAACAAAAATTTAACGCGAAT
                                                                    1625
CCTAAAACGGCTAAAGCCGGATAACCAATTTTTTACTCGACTAAATTGTTTTTAAATTGCGCTTA

TTTAACAAAATATTAACGCTTACAATTTAGGTGGCACTTTTCGGGGAAATGTGCGCGGAACCCCT
                                                                    1690
AAATTGTTTTATAATTGCGAATGTTAAATCCACCGTGAAAAGCCCCTTTACACGCGCCTTGGGGA

ATTTGTTTATTTTTCTAAATACATTCAAATATGTATCCGCTCATGAGACAATAACCCTGATAAAT
                                                                    1755
TAAACAAATAAAAAGATTTATGTAAGTTTATACATAGGCGAGTACTCTGTTATTGGGACTATTTA

GCTTCAATAATATTGAAAAAGGAAGAGTATGAGTATTCAACATTTCCGTGTCGCCCTTATTCCCT
                                                                    1820
CGAAGTTATTATAACTTTTTCCTTCTCATACTCATAAGTTGTAAAGGCACAGCGGGAATAAGGGA

TTTTTGCGGCATTTTGCCTTCCTGTTTTTGCTCACCCAGAAACGCTGGTGAAAGTAAAAGATGCT
                                                                    1885
AAAAACGCCGTAAAACGGAAGGACAAAAACGAGTGGGTCTTTGCGACCACTTTCATTTTCTACGA

GAAGATCAGTTGGGTGCACGAGTGGGTTACATCGAACTGGATCTCAACAGCGGTAAGATCCTTGA
                                                                    1950
CTTCTAGTCAACCCACGTGCTCACCCAATGTAGCTTGACCTAGAGTTGTCGCCATTCTAGGAACT

GAGTTTTCGCCCCGAAGAACGTTTTCCAATGATGAGCACTTTTAAAGTTCTGCTATGTGGCGCGG
                                                                    2015
CTCAAAAGCGGGGCTTCTTGCAAAAGGTTACTACTCGTGAAAATTTCAAGACGATACACCGCGCC

TATTATCCCGTATTGACGCCGGGCAAGAGCAACTCGGTCGCCGCATACACTATTCTCAGAATGAC
                                                                    2080
ATAATAGGGCATAACTGCGGCCCGTTCTCGTTGAGCCAGCGGCGTATGTGATAAGAGTCTTACTG

TTGGTTGAGTACTCACCAGTCACAGAAAAGCATCTTACGGATGGCATGACAGTAAGAGAATTATG
                                                                    2145
AACCAACTCATGAGTGGTCAGTGTCTTTTCGTAGAATGCCTACCGTACTGTCATTCTCTTAATAC

CAGTGCTGCCATAACCATGAGTGATAACACTGCGGCCAACTTACTTCTGACAACGATCGGAGGAC
                                                                    2210
GTCACGACGGTATTGGTACTCACTATTGTGACGCCGGTTGAATGAAGACTGTTGCTAGCCTCCTG
```

```
CGAAGGAGCTAACCGCTTTTTTGCACAACATGGGGGATCATGTAACTCGCCTTGATCGTTGGGAA
                                                                   2275
GCTTCCTCGATTGGCGAAAAAACGTGTTGTACCCCCTAGTACATTGAGCGGAACTAGCAACCCTT

CCGGAGCTGAATGAAGCCATACCAAACGACGAGCGTGACACCACGATGCCTGTAGCAATGGCAAC
                                                                   2340
GGCCTCGACTTACTTCGGTATGGTTTGCTGCTCGCACTGTGGTGCTACGGACATCGTTACCGTTG

AACGTTGCGCAAACTATTAACTGGCGAACTACTTACTCTAGCTTCCCGGCAACAATTAATAGACT
                                                                   2405
TTGCAACGCGTTTGATAATTGACCGCTTGATGAATGAGATCGAAGGGCCGTTGTTAATTATCTGA

GGATGGAGGCGGATAAAGTTGCAGGACCACTTCTGCGCTCGGCCCTTCCGGCTGGCTGGTTTATT
                                                                   2470
CCTACCTCCGCCTATTTCAACGTCCTGGTGAAGACGCGAGCCGGGAAGGCCGACCGACCAAATAA

GCTGATAAATCTGGAGCCGGTGAGCGTGGGTCTCGCGGTATCATTGCAGCACTGGGGCCAGATGG
                                                                   2535
CGACTATTTAGACCTCGGCCACTCGCACCCAGAGCGCCATAGTAACGTCGTGACCCCGGTCTACC

TAAGCCCTCCCGTATCGTAGTTATCTACACGACGGGGAGTCAGGCAACTATGGATGAACGAAATA
                                                                   2600
ATTCGGGAGGGCATAGCATCAATAGATGTGCTGCCCCTCAGTCCGTTGATACCTACTTGCTTTAT

GACAGATCGCTGAGATAGGTGCCTCACTGATTAAGCATTGGTAACTGTCAGACCAAGTTTACTCA
                                                                   2665
CTGTCTAGCGACTCTATCCACGGAGTGACTAATTCGTAACCATTGACAGTCTGGTTCAAATGAGT

TATATACTTTAGATTGATTTAAAACTTCATTTTTAATTTAAAAGGATCTAGGTGAAGATCCTTTT
                                                                   2730
ATATATGAAATCTAACTAAATTTTGAAGTAAAAATTAAATTTTCCTAGATCCACTTCTAGGAAAA

TGATAATCTCATGACCAAAATCCCTTAACGTGAGTTTTCGTTCCACTGAGCGTCAGACCCCGTAG
                                                                   2795
ACTATTAGAGTACTGGTTTTAGGGAATTGCACTCAAAAGCAAGGTGACTCGCAGTCTGGGGCATC

AAAAGATCAAAGGATCTTCTTGAGATCCTTTTTTTCTGCGCGTAATCTGCTGCTTGCAAACAAAA
                                                                   2860
TTTTCTAGTTTCCTAGAAGAACTCTAGGAAAAAAAGACGCGCATTAGACGACGAACGTTTGTTTT

AAACCACCGCTACCAGCGGTGGTTTGTTTGCCGGATCAAGAGCTACCAACTCTTTTTCCGAAGGT
                                                                   2925
TTTGGTGGCGATGGTCGCCACCAAACAAACGGCCTAGTTCTCGATGGTTGAGAAAAAGGCTTCCA

AACTGGCTTCAGCAGAGCGCAGATACCAAATACTGTCCTTCTAGTGTAGCCGTAGTTAGGCCACC
                                                                   2990
TTGACCGAAGTCGTCTCGCGTCTATGGTTTATGACAGGAAGATCACATCGGCATCAATCCGGTGG

ACTTCAAGAACTCTGTAGCACCGCCTACATACCTCGCTCTGCTAATCCTGTTACCAGTGGCTGCT
                                                                   3055
TGAAGTTCTTGAGACATCGTGGCGGATGTATGGAGCGAGACGATTAGGACAATGGTCACCGACGA
```

```
GCCAGTGGCGATAAGTCGTGTCTTACCGGGTTGGACTCAAGACGATAGTTACCGGATAAGGCGCA
                                                                   3120
CGGTCACCGCTATTCAGCACAGAATGGCCCAACCTGAGTTCTGCTATCAATGGCCTATTCCGCGT

GCGGTCGGGCTGAACGGGGGGTTCGTGCACACAGCCCAGCTTGGAGCGAACGACCTACACCGAAC
                                                                   3185
CGCCAGCCCGACTTGCCCCCCAAGCACGTGTGTCGGGTCGAACCTCGCTTGCTGGATGTGGCTTG

TGAGATACCTACAGCGTGAGCTATGAGAAAGCGCCACGCTTCCCGAAGGGAGAAAGGCGGACAGG
                                                                   3250
ACTCTATGGATGTCGCACTCGATACTCTTTCGCGGTGCGAAGGGCTTCCCTCTTTCCGCCTGTCC

TATCCGGTAAGCGGCAGGGTCGGAACAGGAGAGCGCACGAGGGAGCTTCCAGGGGGAAACGCCTG
                                                                   3315
ATAGGCCATTCGCCGTCCCAGCCTTGTCCTCTCGCGTGCTCCCTCGAAGGTCCCCCTTTGCGGAC

GTATCTTTATAGTCCTGTCGGGTTTCGCCACCTCTGACTTGAGCGTCGATTTTTGTGATGCTCGT
                                                                   3380
CATAGAAATATCAGGACAGCCCAAAGCGGTGGAGACTGAACTCGCAGCTAAAAACACTACGAGCA

CAGGGGGGCGGAGCCTATGGAAAAACGCCAGCAACGCGGCCTTTTTACGGTTCCTGGCCTTTTGC
                                                                   3445
GTCCCCCCGCCTCGGATACCTTTTTGCGGTCGTTGCGCCGGAAAAATGCCAAGGACCGGAAAACG

TGGCCTTTTGCTCACATGTTCTTTCCTGCGTTATCCCCTGATTCTGTGGATAACCGTATTACCGC
                                                                   3510
ACCGGAAAACGAGTGTACAAGAAAGGACGCAATAGGGGACTAAGACACCTATTGGCATAATGGCG

CTTTGAGTGAGCTGATACCGCTCGCCGCAGCCGAACGACCGAGCGCAGCGAGTCAGTGAGCGAGG
                                                                   3575
GAAACTCACTCGACTATGGCGAGCGGCGTCGGCTTGCTGGCTCGCGTCGCTCAGTCACTCGCTCC

AAGC     3'
     •••    3579
TTCG     5'
```


Supplementary File 1. Sequence of *gfp* RNAi clone used in this study.
